# Work stress, technological changes, and job insecurity in the retail organization context

**DOI:** 10.3389/fpsyg.2022.918065

**Published:** 2022-10-20

**Authors:** Bilqees Ghani, Khalid Rasheed Memon, Heesup Han, Antonio Ariza-Montes, Juan M. Arjona-Fuentes

**Affiliations:** ^1^Institute of Business Management, Karachi, Pakistan; ^2^Graduate School of Business, Universiti Sains Malaysia, George Town, Penang, Malaysia; ^3^Department of Hospitality and Tourism Management, Sejong University, Seoul, South Korea; ^4^Social Matters Research Group, Universidad Loyola Andalucía, Córdoba, Spain

**Keywords:** job stress, technological changes, job insecurity, retail industry, employees

## Abstract

The study intends to investigate the relationship between work stress and job insecurity, as well as technological changes and job insecurity, with job satisfaction acting as a mediator. The study was conducted among Pakistani retail industry employees using survey questionnaires distributed online and in stores. The sample was composed of 262 retail workers from the FMCG and shopping mall industries. The responses were screened using the statistical software tool SPSS, and hypotheses were examined through SMART-PLS. The findings show that work stress has a strong relationship with job insecurity; additionally, the relationship appears to be statistically significant (β = 55.7%, *p <* 0.05), indicating that there is an increased level of job insecurity if work stress is increased. However, technological advancements showed less influence on job insecurity and had statistically insignificant results (β = 5.9%, *p* > 0.05). This demonstrates that many technological changes cause high levels of job insecurity because employees fear that they will be unable to cope with the changing environment. Furthermore, the mediating mechanism of job satisfaction was found to be significant, as employees with lower levels of satisfaction reported higher levels of insecurity, aiding in the narrowing of the gap in this section of the study. The study also has practical implications because the results show that the retail industry needs to act quickly to make sure workers do not worry about losing their jobs, especially now that COVID-19 is spreading like wildfire.

## Introduction

The retail industry has established strong footholds in major cities, such as Lahore and Karachi, contributing significantly to Pakistan’s overall growth domestic product (GDP). According to the survey conducted by the Pakistan Bureau of Statistics in 2015-16, it was reported that the retail industry is worth $32.89 billion, indicating that the retail industry is contributing around 18.6% of the GDP (Syedain, 2020), and it has been predicted that in the upcoming years, the retail industry of Pakistan will emerge as the fastest-growing retail market, because the majority of Pakistan’s population, around two-thirds, represents the youth (people falling below the age par value of 30), and the increasing income of this particular segment will increase the profitability and popularity of the retail sector. Moreover, the improvement in the infrastructure facilities, which has greatly eased the supply chain process, has been one of the contributing factors to the success of the industry (Faulks et al., 2021). The overall success of the retail industry has given rise to tough competition between firms over the past few decades. Organizations are now striving toward eliminating the cost by following Porter’s cost leadership strategy by reducing payroll expenses and opting for downsizing (Guthrie, 2008; Alsharif et al., 2021). Downsizing is characterized as a management strategy to vacate positions or jobs to improve organizational efficiency and profitability (Marques, 2014). According to Guthrie (2008) and Niesen and Hootegem (2018), this concept has a significant impact on organizational culture and employee relations, which reflects that downsizing is the major cause of job insecurity and harms individual and organizational performance (Guthrie, 2008; Niesen and Hootegem, 2018). Job insecurity, on the other hand, is a perceived feeling of fear about losing one’s job, as well as one of the major reasons why an employee feels stressed about their respective jobs (Vijayan, 2017). Furthermore, as firms are now required to streamline their operations through technological means, globalization has posed many challenges for industries. However, the coronavirus (COVID-19) pandemic has emerged as the decade’s most serious challenge. It has influenced people’s lifestyles and exerted a negative impact on their health, social, and financial situations, since it undoubtedly requires the employee to gain a strong grasp on how to operate those machines and software, which may pose a problem for other employees who are more comfortable complying with their traditional ways of performing their jobs, thus increasing job insecurity (Carnevale, 2020).

Therefore, job insecurity has become the industry’s top concern. Retailers are known for turning over their staff regularly, and as a result, they place a priority on finding and keeping talented individuals. Employees in this top sector are starting to feel insecure about their careers. Employees have experienced negative mental stress as a result of their workload and working environment, which has increased their sense of job insecurity (Lazarus and Folkman, 1984; Guberina and Wang, 2021). According to Soelton et al. (2020), job insecurity causes stress-related mental disorders. Stress can be caused by many things, such as a company’s maximum goal, work environment, or a salary that does not meet your needs. Stress in the workplace can cause great losses, especially for companies. Furthermore, as technology advances, so does the retail industry (Brougham and Haar, 2020). Adapting to new technology is difficult and requires both physical and mental effort (Torous et al., 2020). People feel less secure in their jobs because of the manner in which technological advances have changed the perception of jobs. Given that at the time of COVID-19 and post-COVID-19, the upcoming predicted changes in jobs, work, and employment due to technological revolutions, such as robotics and artificial intelligence, as well as economic fluctuation and political inferences, may also affect employees’ psychological aspects and cause job insecurity (Nam, 2019). In a nutshell, work stress and changes in technology are linked to bad things happening to employees. Job insecurity is one of the main things that can hurt the growth and productivity of an organization.

Following a pandemic, health professionals, scientists, and managers typically begin focusing on the virus and other biological risks involved, advising disease prevention and treatment measures (Tucci et al., 2017). The psycho-economic (psychological and economic) consequences (e.g., job insecurity) among retail workers during a pandemic are frequently ignored or underestimated, resulting in hampering the retail industry growth. Researchers believe that the COVID-19 pandemic can cause negative economic and psychological buffers, leading to feelings of insecurity (Ornell et al., 2020). As a result, we believe it is critical to understand the potential psycho-economic causes of job insecurity or fear of losing a job during a pandemic.

In the past, a large number of studies have mostly looked at the association of job insecurity with different organizational and individual outcomes, such as job performance, employees’ health, work stressors, and employees attitudes and behaviors (Mauno et al., 2001; Sora et al., 2010; Virtanen et al., 2011; Bohle et al., 2018). One more study used the longitudinal methodological approach to examine the relationship between job insecurity and employees’ wellbeing in a Western context, namely, Finland (Mauno and Kinnunen, 1999). Sarwar et al. (2021a) investigated the impact of job insecurity on employees’ psychological wellbeing in the hotel industry during the COVID-19 pandemic. A recent study (López Bohle et al., 2021) found a link between job insecurity and employee affect and procedural justice. Cuyper et al. (2008) contributed to the research on job insecurity by incorporating it as a variable between engagement and life satisfaction. Furthermore, Abbas et al. (2021) found that job insecurity is positively related to economic deprivation and negatively related to mental health, self-esteem, life satisfaction, and economic self-efficacy among hospitality workers. A longitudinal study looked at job insecurity as a risk factor for organizational brittleness, psychological stress and burnout, and organizational withdrawal (Dekker and Schaufeli, 1995). Sora et al. (2019) used the factor, i.e., temporary employment, as an indicator to measure job insecurity and predict the affective wellbeing triggered by job insecurity. A couple of studies have also examined job insecurity in the context of technological changes in the hotel industry (Jung et al., 2021; Koo et al., 2021). Brougham and Haar (2018) and Shankar et al. (2021) investigated changes in the retail context as a result of advanced technology. Furthermore, because there must be social or psychological factors to clearly establish and explain the relationships, the effect of intervening buffers must be considered in order to comprehend the variability in employee reactions (Aguiar-Quintana et al., 2021; Chen and Eyoun, 2021). Various intervening factors have been investigated to strongly predict job insecurity. A study looked at the relationship between job strain and job insecurity through the lens of job pressure (Strazdins et al., 2004), while Aguiar-Quintana et al. (2021) investigated the effect of psychological strain on job insecurity and job performance. Another study conducted in Saudi Arabia found that job insecurity is related to financial anxiety through the mediating effect of work-related flow (Basyouni and El Keshky, 2021). Vo-Thanh et al. (2020) state that perceived health risk due to COVID-19 is linked to perceived job insecurity in a way that makes job performance worse.

The current study, on the other hand, aims to determine the relationships between work-stress-job insecurity and technological changes-job insecurity in the retail context, particularly during COVID-19. Nevertheless, various studies have been conducted to examine job insecurity in response to work stress and technological disruption in the hotel and tourism industry (Velnampy and Niresh, 2012; Jung et al., 2021; Sarwar et al., 2021b; Vo-Thanh et al., 2022). The retail context is largely being overlooked to investigate the issue of employees’ job insecurity in the COVID-19 times. Existing literature, however, greatly supports the evidence that work stress and technological disruption/changes will be positively correlated with job insecurity in the pandemic of COVID-19 (Shin and Hur, 2019; Wang et al., 2021; Rajthilak et al., 2022). Based on literature evidence, a few major studies on job insecurity are summarized in Table 1. Literature also suggests that job satisfaction is the link between work stress and job insecurity, as well as between job insecurity and technological changes (Yeves et al., 2019; Jung et al., 2021).

**TABLE 1:** Respondents’ profile.

		Frequency	Percentage	Cumulative percentage
**Age**	20–30	132	50.4	50.4
	31–40	113	43.1	93.5
	41–50	15	5.7	99.2
	51–60	2	0.8	100.0
	Total	262	100.0	
**Gender**	Female	99	37.8	37.8
	Male	163	62.2	100.0
	Total	262	100.0	
**Education**	Matric	6	2.3	2.3
	Intermediate	48	18.3	20.6
	Bachelors	83	31.7	52.3
	Masters	120	45.8	98.1
	M.Phil/Ph.D	5	1.9	100.0
	Total	262	100.0	

Finally, our study aims to examine the perception of retail industry employees regarding their job insecurity in the era of pandemic COVID-19. The COVID-19 pandemic has been marked by increased depression, anxiety, and consequently, job insecurity. This may be due to the impact on economic and labor market performance. In COVID-19, retail employees experienced the highest level of job insecurity (i.e., permanent loss of a job or loss of job features). Recessions increase the risk of poor mental health outcomes. During the excessive recession, job-related impacts (e.g., lost a job, took a job below experience, and took on additional job) were linked to anxiety and depression (Wilson et al., 2020).

**FIGURE 1:**
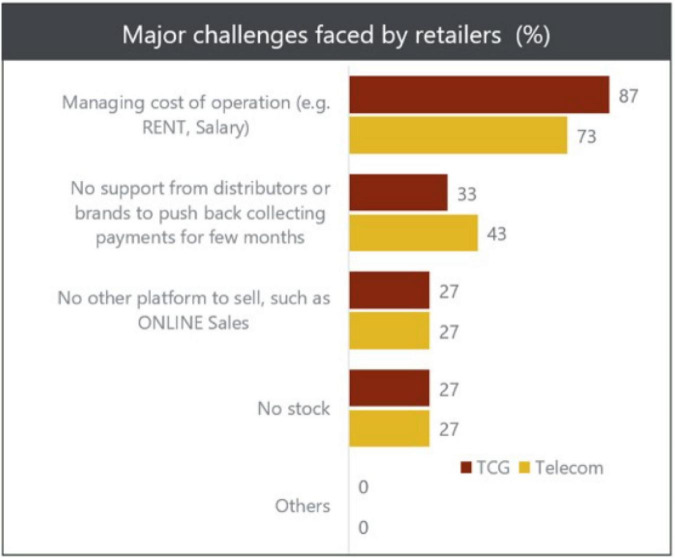
COVID-19 Impact on Retailer Sentiments in Pakistan, 2020.

The graph below (refer Figure 1) shows that due to the COVID-19 outbreak, retail store sales fell from positive growth of 3.11% to a negative growth of 0.31% in 2019–2020. This resulted in the hampering of sales of the retail outlets due to the social distancing and lockdown situations. People came up with the idea of avoiding shopping at the retail outlets and shifted their buying patterns from impulsive buying to buying essentials only, which hence resulted in a deterioration of overall sales at the retail outlets, according to the survey conducted by GfK across the six major cities of Pakistan, i.e., Karachi, Lahore, Islamabad, Faisalabad, Peshawar, and Multan. The major challenge faced by 80% of the retailers is managing the operational costs, which include salaries and payrolls (Growth from Knowledge [GfK], 2021).

**FIGURE 2:**
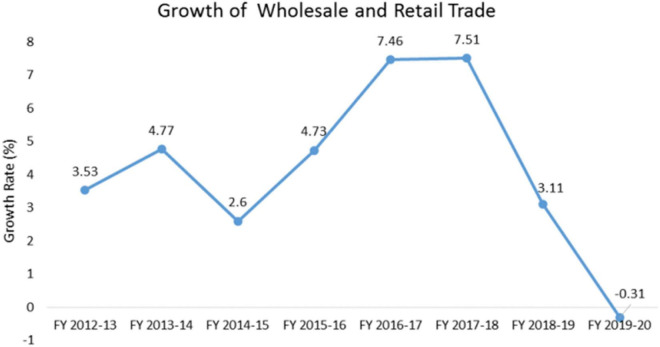
Covid19 and Innovation in Retail- Pakistan’s Growth Industry, 2020. Estimated model after bootstrapping.

The below chart (refer Figure 2) also explains the decline in growth of the retail sector over the past years from 2017 to 2018.

The study explains the relationship between work stress and job insecurity, as well as between technological changes and job insecurity underpinned by social exchange theory. According to the social exchange paradigm, when employees exhibit a high level of stress, their level of job insecurity increases. Employees also perceive job insecurity when technological changes are underway. Furthermore, the effect of the intervening mechanism must be considered in order to comprehend the variability in employee reactions. By using job satisfaction as a mediator, the study also aims to fill in a gap and make a strong link between work stress and job insecurity and between technological changes and job insecurity.

## Theoretical framework and hypotheses development

For our research, we chose the social exchange theory to support our study conceptual link between work stress and job insecurity, and between technological changes and job insecurity. The interactions and relationships between people are critically based upon social exchange behavior, which is highlighted by Cook and Gerbasi (2012) as the reciprocity norm, which certainly states that two parties are interdependent and a positive attitude or behavior of one party automatically obligates the other party to reciprocate the same treatment in return. Therefore, people measure the cost and benefit of a relationship. This evaluation of cost and benefit determines whether to continue the relationship or abandon it. Social exchange theory provides a paradigm for predicting key HR functions, such as employee commitment, employee motivation, and organizational satisfaction and loyalty (Cherry, 2020).

## The relationship between work stress and job insecurity

Job insecurity is becoming more relevant in the study of occupational health as a result of the global economic crisis. Work-related stress (WRS) is a complex phenomenon in general and appeared more challenging during the COVID-19 pandemic. Work stress develops when multiple psychosocial risk factors coexist and interact. The psychosocial risks that influence the perception of stress are usually driven by excessive workloads, a lack of decisional autonomy in the management of one’s work, the presence of relational conflicts in the workplace, and more importantly, fear of losing a job. These stressors suffer employees a lot because they make people feel uncertain about their jobs (De Sio et al., 2018; Menéndez-Espina et al., 2019). According to one study, people’s job insecurity is very low when they are under low job pressure. High job pressure has an impact on a person’s mental and physical health. According to the study’s findings, lower job pressure is associated with lower job insecurity and moderate strain (Strazdins et al., 2004). This mechanism is also supported by social exchange theory, because the influence of work stress on job insecurity is based on a psychological contract (Piccoli et al., 2017). Because our behavior is based on our psychological contract perception, we believe that employees’ job insecurity increases as their work stress increases. From the standpoint of social exchange theory, employee behavior in the context of job insecurity is typically driven by concerns about the exchange of organizational resources, i.e., work stress (Costa and Neves, 2017). Work stress or work pressure sometimes makes a person think he is not capable of doing a task. Work stress seemed to be very frustrating for several people, which made them quit their jobs or feel that they might be fired from the company. It leads to poor health and mental disturbance in an employee and creates job insecurity as a result (Llosa et al., 2018). As a result, we conclude that an increase in work stress will lead to a perception of increased job insecurity among retail industry workers. As a result, we propose our first hypothesis:

H1: *Work stress is positively related to job insecurity such that an increase in work stress increases job insecurity and a decrease in work stress decreases job insecurity.*

## The relationship between technological changes and job insecurity

Even before COVID-19, it was clear that modern workplaces face uncertainty as a result of technological changes, making it impossible to guarantee employment stability to all employees. Employee perceptions of job insecurity have risen as a result of new technologies, but COVID-19 has made things worse (Etehadi and Karatepe, 2019). The stability of a job is a major concern for an employee these days due to technological advancement. Job insecurity is becoming a strong stressor in the current era. Organizational contexts are changing day by day, and so are employment situations, with their resultant impact on the determination of job security (Lee et al., 2017). Globally, technology has a significant impact on job quality determination. Automation and artificial intelligence have made technology a substitute for human labor. The impact of technological changes has greatly increased the job insecurity factor. Technology is not only making life easier for businesses, but it is also increasing job insecurity among employees. This has also made workers stressed out and hurt their mental health (Schulte and Howard, n.d.). COVID-19 has shifted everything to online mode, which means new technological advancements and skill requirements have been incorporated into work practices (Sahni, 2020). The majority of employees observed a lack of awareness of new technology. Typically, the middle-aged group or senior employees over 50 years of age have faced the most disadvantages, resulting in an increase in job insecurity as a result of technological advancement. The employee perception was observed with an increase in concerns like the inability to fit in with new technology and work comfortably (Shankar et al., 2021). The social exchange theory is observed for its role in explaining the responsiveness factor among employees toward technological changes and its input in the determination of the level of job insecurity. The social exchange theory explains the association of technology as an element of irritation among traditional employees to come up with an increase in job insecurity. In contrast, the study also found that the role of technological changes among young employees was positively associated with a decrease in job insecurity (Wang et al., 2019). The treatment of job insecurity was reported with proper training of the new system and making the employees aware of it. Hence, we suggest the following hypothesis:

H2: *Technological changes are positively related to job insecurity such that an increase in technological changes increases job insecurity and a decrease in technological changes decreases job insecurity.*

## Mediation of job satisfaction in the relationship between work stress and job insecurity

Work stress is the most significant organizational issue influencing employees’ job satisfaction. Job satisfaction is a positive and pleasant emotional state that results from an individual’s evaluation of his or her job or job experience. When employees’ needs are not met at work or they face the fear of losing their job, they are more likely to experience a high level of job stress, which can negatively impact their level of job satisfaction (Hosseinabadi et al., 2018). A study conducted at a well-known IT firm and a government agency in Texas, USA, showed a strong correlation between work stress and job satisfaction (McCalister et al., 2006). Another study revealed the transactional model of stress and the person-environment (P-E) fit model (Buitendach and De Witte, 2005). We use a social exchange theory to describe that work stress-related factors, including supervisor support, play a big role in job satisfaction. The relationships between employer and employee are based on an exchange perspective. When employees perceive low work stress from their employer, i.e., low strain in terms of uncontrollability and unpredictability, they show high job satisfaction (Ali, 2021). In another research conducted in the US among college students of 14 universities having retail work experience, similar results were seen where students who had less work stress reported higher levels of job satisfaction; a high level of job satisfaction then leads to a low level of job insecurity (Kim et al., 2009).

Furthermore, previous research on job satisfaction suggested that employees who believe they are safe and do not fear losing their job may be less stressed at work. People who are happy at work are less likely to be concerned about layoffs because they have a psychologically stable foundation. As a result, they tend to develop a high level of expertise, resulting in better job performance. In other words, job satisfaction is a primary source of feeling a secure job (Kim and Kim, 2020). We appeal to a social exchange theory with a psychological contract perspective, particularly in the domain of job insecurity literature, since it refers to the reciprocal expectations held by employees regarding their workplace obligations (Buitendach and De Witte, 2005). When employees feel happy and comfortable both physically and psychologically, this shows their higher job satisfaction; in return, they feel secure themselves and reduce their behavior of job insecurity (Dennick and Tavakol, 2011). This shows that an increase in work stress is inversely related to job satisfaction, such as when an increase in work stress causes a decrease in job satisfaction, which leads to increased job insecurity. Based on such evidence, it can be shown that job satisfaction plays an intervening role between work stress and job insecurity. The discussion eventually led to the conclusion that while work stress has a significant impact on job insecurity, the cause of the increase in job insecurity is mediated by the factor of job satisfaction. We, therefore, formulate the following hypothesis:

H3: *Job satisfaction mediates the relationship between work stress and job insecurity.*

## Mediation of job satisfaction in the relationship between technological changes and job insecurity

In a study of 396 Australian pharmacists, the relationship between technological changes and job satisfaction, including other variables, was studied. A comparison of pharmacists with other professionals in different fields was also performed. In the results of the study, it was concluded that due to technological changes, pharmacists showed a lower level of job satisfaction. The results also showed community pharmacists having an even lower score than hospital pharmacists, showing that an unorganized structure will face more problems with employees when making technological changes (Humphrys and O’BRIEN, 1986). Another study conducted in Mexico about technological changes in a workplace with generation X and generation Y employees found that intrinsic motivation was on the lower side for generation X, resulting in lower job satisfaction, whereas generation Y was deemed to be more adaptive to new technology (Elias et al., 2012). According to a study finding, technological changes in the workplace and advancements are intended to help employees work more effectively and productively. However, employees may become more frustrated and subsequently less satisfied with their job due to the extent to which they might feel afraid of not being comfortable with the changes in technology, since some workers could suffer from their inability to translate previous experience into the technological ground, an issue that might hurt their performance or reduce their satisfaction levels at work (Brumberg, 2018).

The increase in job satisfaction also shows a decrease in job insecurity. From the social exchange perspective, with the increase in downsizing and restructuring, many employees have shown lower levels of job satisfaction and higher levels of job insecurity, which indicates the fear of being laid off or permanently losing their job. If organizations can adapt to such practices where work stress and technological changes are low and job satisfaction is high, then they will also see that this job satisfaction leads to less fear among employees of losing their job and lower levels of job insecurity. For example, dealing with difficult customers on a daily basis causes dissatisfaction and stimulates negative emotional feelings. Over time, employees’ anxiety levels will rise as a result of such negative feelings and workplace unhappiness, i.e., job dissatisfaction. Employees who are dissatisfied with their jobs as a result of radical technological changes may experience anxiety, perceive different work situations as potentially threatening, and feel less secure about their job. As a result, we believe that increased job satisfaction will reduce job insecurity. Overall, technological disruptions decrease job satisfaction, which increases job insecurity. As a result, job satisfaction acts as a bridge between technological change and job insecurity (Cheung et al., 2019).

H4: *Job satisfaction mediates the relationship between work stress and technological changes.*

**FIGURE 3:**
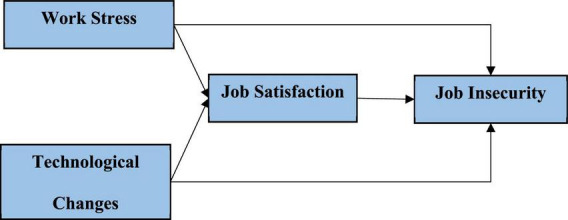
Conceptual framework.

Based on these hypotheses, a conceptual framework has been developed, which is depicted in Figure 3.

## Methodology

### Sample and procedure

The study is based on a quantitative research approach with the purpose of investigating the relationships between work stress and job insecurity, and technological changes and job insecurity with the mediating effect of job satisfaction. Since the study is cross-sectional in design, the responses were collected from the targeted population using a questionnaire as a data collection instrument and a survey as a data collection approach (Alsharif et al., 2021). The participants of the survey were chosen through the convenience sampling method because the samples were readily available and for the quick collection of the data (Question Pro, 2021). Karachi shopping malls and FMCG stores were visited, including Dolmen City Mall (Clifton), Dolmen Mall (Tariq Road), Luckyone, Bin Hashim (Safoora), Bin Safeer, and Imtiaz Super Store (Gulshan). The estimated targeted population was around 4,100. The sample size was determined using the G-Power tool (VanVoorhis and Morgan, 2007). The sample size N was calculated by the priori power analysis (Cohen, 1988) based on the suitable degree of power (1-β), the pre-specified degree of significance, and the magnitude of the population effect that may be seen with probability (1-β). Before a thorough study is done, *a priori* analysis can regulate the prediction potential effectively (Hager, 2006; Faul et al., 2007). The minimum sample size was found to be 107, and actual power was 85% when using the program G*Power 3 with the following input parameters: medium effect, probability of Type I error α = 0.05, and probability of Type II error β = 0.05, which means (1-β) = 0.95 and number of predictors = 2. Thus, the sample size of 262 is enough to justify our results. The same method has been used by several previous studies (see, e.g., Ooi et al., 2020; Memon and Ghani, 2021; Memon et al., 2021a,b).

The employees chosen were the survivors of organizational restructuring in the era of COVID-19, particularly in the retail industry. The inclusion criteria are as given below. The respondent must be an employee of a retail store; the employee must have more than 2 years of experience with the store; and the respondent must be a front-line employee. The researcher surveyed by providing the questionnaire to retail employees, explaining the purpose of the study and collecting responses by visiting their stores two and three times, which also resulted in an increased response rate. The key feature was standardized questions to ensure the compatibility of the questions. Accordingly, the data were collected by circulating 380 questionnaires, while completely filled responses in all aspects were 262.

### Instruments/measures

All instruments were adopted with established reliability and validity. A 5-point Likert scale was used to evaluate the answers ranging from strongly agree to strongly disagree.

#### Work stress

The scale was adopted from a previous study (Sarwar et al., 2020). Cronbach’s alpha is 0.855, which is higher than 0.6, so it is good enough to use with the work stress reliability scale. Sample items included in the questionnaire are as follows: “I feel uneasy about losing my job in the near future.” and “I worry a lot about not being able to keep my job.”

#### Technological changes

The scale was adopted from a previous study (Sarwar et al., 2020). Cronbach’s alpha value is.779, and as the value is higher than 0.6, it is sufficient to accept the reliability scale of technological changes. A few instances are as follows: “The automation system is determined to turn the workplace into robot-operated units. I feel insecure that I might lose my job due to this reason. I fear for my job if I don’t learn new skills soon” and “My colleagues who have higher technological skills get higher pay than me.”

#### Job satisfaction

The scale was adopted from a previously published study (Sarwar et al., 2020). Cronbach’s alpha value is.767, and as the value is higher than 0.6, it is sufficient to accept the job satisfaction reliability scale. A few sample items are as follows: “Taking everything into account, I am satisfied with my job” and “In this organization, I can prevent negative things from affecting my work situation.”

#### Job insecurity

The scale was adopted from a previously published study (Sarwar et al., 2020). Cronbach’s alpha value is.836, and as the value is higher than 0.6, it is sufficient to accept the reliability scale of job insecurity. A few sample items are as follows: “I feel my depression has increased my job insecurity” and “I feel my irritableness has increased my job insecurity.”

### Demographic information

The demographic information of the respondents is presented in Table 1. It was shown that the response rate was approximately 68.9% and the majority of the respondents were in the age bracket of 20–40 years old, at around 73.5%. The gender-based composition of respondents was observed with a majority of male respondents. When respondents’ education levels were taken into account, it was found that about 77% of respondents had a bachelor’s degree or higher.

### Common method variance assessment

Harman’s single-factor test has been used to find problems with common method variance. The first and greatest component only accounted for 36.50% of the variation, which is below the 50% threshold that was determined by the research. This means that there is no single element that is responsible for the vast majority of the variation in the data. In this instance, we may rest comfortably that the issue of common method variance is not significant (Podsakoff et al., 2012).

### Descriptive statistics

Descriptive statistics were used (Table 2) to measure the central tendency of the data, as shown below. Having a large number of extreme values in a dataset might lead to a skewed distribution. Things will return to normal once the data is cleaned up. Errors in computation, data input, and outliers are all part of this process, and they must be removed from the data in order to serve a legal purpose. Data distribution is normal, as shown in Table 2, with all the values falling within the normality limits.

**TABLE 2:** Descriptive statistics.

	N	Minimum	Maximum	Mean	SD	Skewness		Kurtosis	
							
	Statistic	Statistic	Statistic	Statistic	Statistic	Statistic	Std. error	Statistic	Std. error
Job insecurity	262	1.00	5.00	3.1282	0.90332	-0.418	150	-0.552	300
Work stress	262	1.00	5.00	3.1807	0.85497	-0.531	150	-0.044	300
Technological changes	262	1.57	5.00	3.5453	0.72581	-0.149	150	-0.439	300
Job satisfaction	262	1.40	5.00	3.0603	0.80717	0.088	150	-0.694	300
Valid *N* (listwise)	262								

### Tools’ assessment (confirmatory factor analysis)

It is essential to examine the validity of measuring instruments in a developing country because most of them have been borrowed from Western research (Ooi et al., 2020). A CFA has been used to assess the validity of all the research instruments in terms of convergent and discriminatory validity. It appears to be an excellent model fit, as shown in Table 3, with all factor loadings meeting the requirements. According to Hair et al. (2014, 2017), factor loading values more than 0.65 were judged good, while those greater than 0.60 were regarded acceptable. As a result, all important values demonstrate an excellent model fit, with NFI = 0.925, TLI = 0.923, AGFI = 0.8278, GFI = 0.875, and RMSEA = 0.058. When evaluating a model’s goodness of fit, a value more than or equal to 0.80 is considered acceptable for the Adjusted Goodness of Fit Index (AGFI), whereas a value less than or equal to 0.09 is deemed acceptable for the RMSEA Goodness of Fit Index (Hair et al., 2014, 2017).

**TABLE 3:** Confirmatory factory analysis (CFA).

Model fit indices	Chi-square	DF	CMIN/DF	GFI	AGFI	NFI	TLI	RMSEA
	668.254	300	2.652	0.875	0.908	0.925	0.923	0.058

DF, Degrees of Freedom; CMIN/DF, minimum discrepancy; GFI, Goodness of Fit Index; AGFI, Adjusted Goodness of Fit Index; NFI, Normed Fit Index; TLI, Tucker–Lewis Index; RMSEA, Root Mean Square Error of Approximation.

### Validity and reliability tests

The research measured the structural model and the measurement model using SMART-PLS 3. Each variable was evaluated using Fornell and Larcker’s (1981) approach to mean extracted variance (AVE). Accordingly, there were factor loadings above 0.7, average extracted variance (AVE) above 0.5, and composite reliability (CR) above 0.7, and the conditions for convergent validity were fulfilled (Fornell and Larcker, 1981). As for reliability, Cronbach’s alpha values (Cronbach, 1951) are much over the threshold value of 0.7 (Nunnally, 1978). The reliability and convergent validity of all variables are shown in Table 4.

**TABLE 4:** Construct reliability and convergent validity through AVE.

Construct	Cronbach’s alpha	No. of items	AVE	CR
Job insecurity	0.836	5	0.756	0.843
Work stress	0.855	6	0.721	0.864
Technological changes	0.779	7	0.745	0.812
Job satisfaction	0.767	5	0.782	0.802

To round things off, discriminant validity technique through HTMT ratio is shown in Table 5. According to Henseler et al. (2014) and Hair et al. (2017), the discriminant validity of a notion may be judged by whether or not its HTMT value is less than 0.85. It was observed that the HTMT score for all constructs was less than or equal to 0.85, showing that the constructs are within an acceptable range. Furthermore, the cross-loadings of all the items were also evaluated to demonstrate discriminant validity. Specifically, each important variable had cross-loadings over 0.7, which was suitable and consistent across all relevant variables.

**TABLE 5:** Discriminant validity (HTMT ratio).

	Work stress	Technological changes	Job satisfaction	Job insecurity
**Work stress**				
**Technological changes**	0.293			
**Job satisfaction**	0.341	0.406		
**Job insecurity**	0.355	0.418	0.545	

### Bootstrapping: Path coefficients

A regression test was conducted *via* SMART-PLS 3 to make hypothesis testing possible, as the model has to be measured for the impact that causal variables of work stress and technological changes have on job satisfaction and, subsequently, on job insecurity. The bootstrapping approach with 5,000 samples and the single *t*-test were used since the direction of the relationships was known. Table 6 shows that there is a significant relationship between work stress and job satisfaction with a beta coefficient value of -0.229 and a *p*-value of 0.005. Similarly, there is also a significant relationship between technological changes and job satisfaction with a beta coefficient value of -0.185 and a *p*-value of 0.002. The study also found a significant relationship between work stress and job insecurity with a coefficient value of 0.316 and a *p*-value of 0.000. It is also observed through the study that there is a positive beta coefficient value of technological change of 0.449 with a *p*-value of 0.000. Finally, job satisfaction was also found to have a beta coefficient value of -0.192 with a *p*-value of 0.005.

**TABLE 6:** Path coefficient (direct effects).

	Beta values	SD	*T*-statistics	*P-*values
Work stress→Job satisfaction	–0.229	0.498	4.428	0.005
Technological changes →Job satisfaction	–0.185	0.573	2.874	0.002
Work stress→Job insecurity	0.316	0.669	3.481	0.000
Technological changes→ Job Insecurity	0.449	0.756	4.142	0.000
Job satisfaction→Job insecurity	–0.192	0.861	6.546	0.005

**FIGURE 4:**
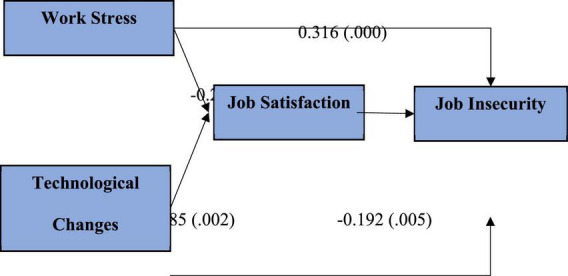
Estimated model after bootstrapping.

The results also measured the predictive accuracy, represented by the value of R square, and it is 31% for adjusted *R*^2^ representing a good model fit. In addition to the aforementioned test, another test was run to compute *F*^2^ (F square values, which measure the contribution of individual variables to R square). For a low contribution, F square values should be at least 0.02; for a moderate contribution, F square values should be larger than 0.15; and for a high contribution, F square values should be greater than 0.35. Our data indicated that F square values for all variables were more than the threshold value, indicating that all factors contributed to R square (Cohen, 2013). Finally, the results of bootstrapping are summarized and represented in Figure 4 showing the beta coefficient values outside brackets and *p*-values inside the bracket.

### Mediating analysis

Table 7 summarizes the mediation analysis results. A bootstrapping technique was used to assess the impact of both independent variables on the dependent variables through the mediating variable job satisfaction. Both independent variables (work stress and technological changes) have significant relationships with beta coefficient values of 0.164 and 0.129. However, partial mediation occurs because the values of the beta coefficient in direct relationships (Table 6) are greater than the mediating effects (Hair and Sarstedt, 2021). Therefore, the direct effect would have a greater impact on the dependent variable of Job Insecurity.

**TABLE 7:** Mediation analysis (indirect effects).

	Beta values	SD	*T*-statistics	*P*-values
Work Stress→Job Satisfaction→ Job Insecurity	0.164	0.982	5.832	0.003
Technological changes →Job Satisfaction→Job Insecurity	0.129	0.893	4.742	0.005

## Discussion

Our first hypothesis has been formulated and tested to see the relationship between work stress and job insecurity. The results show that work stress affects job insecurity by 55.7%. The influence that the causal variable of work stress has on job insecurity proves to be significant since *P* < 0.05. Therefore, we fail to reject the hypothesis, and it can be deduced that there is a positive, significant influence of work stress on job insecurity. This also proves that in the race to become the leading retail store in Pakistan, companies put high pressure on their employees with little to no incentives, which adds to their wellbeing as well, causing illnesses and employees becoming insecure about not being able to perform and losing their job (Sarwar et al., 2020). Another investigation also revealed the positive effect of work stress on the determination of job insecurity. The study significantly explained the relationship between work stress and work insecurity among employees in the service sector (Hai and Rabenu, 2018). Another study also found that the bad effects of work stress lead to less job security (Costa and Neves, 2017). Research suggests that organizations undergo various strategically important decisions, which involve mergers and acquisitions, along with layoff decisions and job sharing among the part-timers, which often lead to work stress and ultimately job insecurity (Niesen and Hootegem, 2018). In addition, the downsizing of survivors’ workload significantly increases, and uncertainty regarding job tasks is observable (Soelton et al., 2020).

The results of our second research objective show that technological changes affect job insecurity by 5.9%. However, the influence that the causal variable technological changes have on job insecurity proves to be insignificant since *P* > 0.05. Therefore, we reject the hypothesis, and it can be deduced that there is no significant influence of technological changes on job insecurity. This shows that the initial fear caused by the changes in technology during COVID-19 has been greatly reduced because most companies use the new technology often and train their employees on how to use it (Torous et al., 2020). On the other hand, the growing need for employees to be technologically equipped has often caused job insecurity among older workers, who often face difficulty in complying with it, thus ultimately giving birth to job insecurity as their employment stability is no longer guaranteed due to an increase in work stress (Etehadi and Karatepe, 2019). According to the results of a study, technological advancements in general are rendering many well-known jobs obsolete. The subsequent wave of economic upheavals would not originate abroad. It will happen because automation is moving so quickly and taking away so many good middle-class jobs (Lee et al., 2018). Also, previous studies on job insecurity only looked at how it affected employees’ health and wellbeing, and they found that work stress made job insecurity worse (Brougham and Haar, 2020).

The results of our third and fourth research objectives showed that work stress and technological changes are inversely related to job satisfaction, i.e., an increase in work stress and technological changes causes a decrease in job satisfaction, and a decrease in job satisfaction leads to increased job insecurity. Many employees felt stressed and were not able to immediately adapt to the technological changes, which caused a low job satisfaction rate and eventually increased their job insecurity (Adiguzel and Kucukoglu, 2019). One of the main causes of work stress is work pressure, a topic of discussion that has intensified among academics and professionals. Workplace pressure may be connected to a number of diseases with ill-defined causes, such as chronic fatigue and overwork. In this situation, handling work pressure improperly could have negative effects on mental health and even lower employee levels of job satisfaction, which leads to increased job insecurity. The high-tech sector is a high-pressure one and could also be the cause of lower job satisfaction and feelings of insecurity at work. However, identifying the main sources of pressure and reducing them would have a significant positive impact on job satisfaction and productivity (Huang and Wang, 2019).

Since business competition is getting tougher in the current era due to globalization and technological advancement, it is difficult for most people to cope with such situations, i.e., unfit job dynamics and the adaption of new technology. This ultimately triggers their psychological stress and creates technological disruption, which causes dissatisfaction in their workplace. This makes them more likely to act in ways that make their jobs less secure (Masia, 2010; Cherry, 2020).

Another study showed evidence that when employees face various workplace threats and fear of adaptations of new technology that are beyond their comfort zone, they may experience high levels of anxiety and discomfort (Kim and Kim, 2020). We speculate that heightened anxiety, i.e., stress and not feeling comfortable due to new technology, leads to reduced job satisfaction and consequently increases job insecurity (Buitendach and De Witte, 2005; Brougham and Haar, 2020).

The most important limitation observed in this study is data collection constraints, as primary data-based analysis is difficult to perform in developing countries. Most importantly, it is hard to talk to employees in an organization because the topic of the current study is related to the organization’s policy input. This makes it hard to talk to staff and get a response. Another limitation is that time management was also found to be a challenge for the present study with a busy work schedule.

## Conclusion and managerial implications

During the COVID-19 pandemic, where employees are insecure about losing their job, it is the responsibility of the management to ensure measures that employees do not feel insecure or demotivated about their job. One way to lower job insecurity levels, as well as work stress, is by introducing digital therapy programs in the organization (Sarwar et al., 2020; Torous et al., 2020; Obrenovic et al., 2021), where employees can get access to mental health counselors and other health professionals who can help them with dealing with the negative emotions, specifically at this time of the pandemic. This will also help colleagues understand each other and will help each other reduce the stress of their co-workers.

As per the research, job insecurity tends to increase with an increase in technological changes. Therefore, it is the duty of the manager and higher management that employees receive effective training to cope with the new technology being introduced in the company. In addition, they should also ensure that new technology is not introduced too rapidly and employees have time to adjust to the current system and, in effect, are given time to adjust to the new system as well. One of the main reasons for job insecurity is layoffs, which have happened during COVID-19. Companies need to make sure they find other ways to run their businesses, like online grocery shopping, instead of laying off employees to save money, which will make job insecurity less of a problem during the pandemic.

Policymakers are also required to come up with effective and feasible policies, i.e., be emphatic, provide training opportunities, financial benefits, etc. to bring about an improved level of HR policies and uplift employees’ level of confidence. Improved HR policies not only make employees feel more secure in their jobs but also make them more productive. This not only holds the interest of employees in the organization but also helps attract talent. So, this makes employees happier with their jobs and reduces the stress they feel from not knowing if they will have a job tomorrow.

The scope of the present study can be extended to the training and development program and managerial support as antecedents to understand with the extensive view of job satisfaction and its role in the determination of job insecurity among employees in the same sector focusing on retail stores or can be extended to other segments of the economy to understand the implications of the selected variables (Radford et al., 2021).

## Data availability statement

The raw data supporting the conclusions of this article will be made available by the authors, without undue reservation.

## Author contributions

BG, KM, HH, AA-M, and JA-F contributed to conceptualization, formal analysis, investigation, methodology and writing and editing the original draft. All authors contributed to the article and approved the submitted version.
