# Supplementary material for: Work stress, technological changes, and job insecurity in the retail organization context
Source: Front Psychol. 2022 Oct 20;13:918065. doi: 10.3389/fpsyg.2022.918065 (PMC9723883; doi:10.3389/fpsyg.2022.918065)
Supplement: Supplementary file 1 [file Data_Sheet_1.docx]

# Appendix A

Table 1. A Summary of Job Insecurity Studies

| Sr. | Research Title | Findings | Source |
| --- | --- | --- | --- |
| 1 | Impact of COVID-19 pandemic on micro, small, and medium-sized Enterprises operating in Pakistan. | Pakistani supermarkets have adopted a low-cost, automated distribution strategy to compete. This has led to job losses and increased employee insecurity. Job cuts have worsened the job market and made it more difficult for companies to recruit and retain skilled staff. | (Shafi, Liu, & Ren, 2020) |
| 2 | Job insecurity, extrinsic and intrinsic job satisfaction, and affective organizational commitment of maintenance workers in a parastatal. | Job insecurity is a psychological condition in which an employee feels threatened about losing their job, affecting their job engagement and work-life balance. Job insecurity and dissatisfaction were linked in the study. | (Buitendach & De Witte, 2005) |
| 3 | Learning at the workplace and sustainable employability. A multi-source model moderated by age. | With new technologies, globalization transformed work-life balance. This increased a company's technical expertise and required employees to learn new industry norms. This has led to technology-equipped operations and fewer employee requirements, increasing job insecurity in customer service. | (Heijden, 2016). |
| 4 | Job Insecurity: Coping with Jobs at Risk. | The study found a correlation between employee age and attitude toward new technology. Thus limiting their job prospects. In recent years, the tourism and hotel industries in Serbia have experienced massive layoffs and frequent changes in working hours and wages. This increases employee turnover. | (Hartley, Jacobson, Klandermans, & Vuure, 1991) |
| 5 | Job insecurity: Correlates, moderators, and measurement | Another study highlighted two aspects of job insecurity: job loss and anxiety about job loss. Changing work practices have increased employee concerns about job security. | (Borg & Elizur, 1992) |
| 6 | The impact of job insecurity on critical hotel employee outcomes: the mediating role of self-efficacy | COVID-19 has caused unusual crises in almost every industry, including tourism, hotels, and retail, due to a sharp drop in consumer demand, sales volume, and firm profitability. This boosted firm-level risk. In response, the organization downsized. | (Etehadi B., 2019) |
| 7 | Employee adjustment and well-being in the era of COVID-19. | Contagious pandemics and frequent lockdowns have forced people to avoid social interaction and public places, endangering the retail industry, especially the clothing and FMCG sectors. This disrupted the supply chain due to health and safety fears. Thus, a tech-based layoff solution. This boosted job insecurity. | (Carnevale J.B., 2020;116(Aug) |

|  |  | Reporting Item | Page Number |
| --- | --- | --- | --- |
| **Title and abstract** |  |  |  |
| Title | [#1a](https://www.goodreports.org/reporting-checklists/strobe-cross-sectional/info/#1a) | Indicate the study’s design with a commonly used term in the title or the abstract | 1 |
| Abstract | [#1b](https://www.goodreports.org/reporting-checklists/strobe-cross-sectional/info/#1b) | Provide in the abstract an informative and balanced summary of what was done and what was found | 1 |
| **Introduction** |  |  |  |
| Background / rationale | [#2](https://www.goodreports.org/reporting-checklists/strobe-cross-sectional/info/#2) | Explain the scientific background and rationale for the investigation being reported | 2 |
| Objectives | [#3](https://www.goodreports.org/reporting-checklists/strobe-cross-sectional/info/#3) | State specific objectives, including any prespecified hypotheses | 4 |
| **Methods** |  |  |  |
| Study design | [#4](https://www.goodreports.org/reporting-checklists/strobe-cross-sectional/info/#4) | Present key elements of study design early in the paper | 11 |
| Setting | [#5](https://www.goodreports.org/reporting-checklists/strobe-cross-sectional/info/#5) | Describe the setting, locations, and relevant dates, including periods of recruitment, exposure, follow-up, and data collection | 12 |
| Eligibility criteria | [#6a](https://www.goodreports.org/reporting-checklists/strobe-cross-sectional/info/#6a) | Give the eligibility criteria, and the sources and methods of selection of participants. | 11 and 12 |
|  | [#7](https://www.goodreports.org/reporting-checklists/strobe-cross-sectional/info/#7) | Clearly define all outcomes, exposures, predictors, potential confounders, and effect modifiers. Give diagnostic criteria, if applicable | 4, 5, 6, 7, 8, 9, & 10 |
| Data sources / measurement | [#8](https://www.goodreports.org/reporting-checklists/strobe-cross-sectional/info/#8) | For each variable of interest give sources of data and details of methods of assessment (measurement). Describe comparability of assessment methods if there is more than one group. Give information separately for for exposed and unexposed groups if applicable. | 12 |
| Bias | [#9](https://www.goodreports.org/reporting-checklists/strobe-cross-sectional/info/#9) | Describe any efforts to address potential sources of bias | 11 |
| Study size | [#10](https://www.goodreports.org/reporting-checklists/strobe-cross-sectional/info/#10) | Explain how the study size was arrived at | 11 & 12 |
| Quantitative variables | [#11](https://www.goodreports.org/reporting-checklists/strobe-cross-sectional/info/#11) | Explain how quantitative variables were handled in the analyses. If applicable, describe which groupings were chosen, and why | 11 & 12 |
| Statistical methods | [#12a](https://www.goodreports.org/reporting-checklists/strobe-cross-sectional/info/#12a) | Describe all statistical methods, including those used to control for confounding | 12, 13, 14, 15, & 16 |
| Statistical methods | [#12b](https://www.goodreports.org/reporting-checklists/strobe-cross-sectional/info/#12b) | Describe any methods used to examine subgroups and interactions | 12, 13, 14, 15, 16 |
| Statistical methods | [#12c](https://www.goodreports.org/reporting-checklists/strobe-cross-sectional/info/#12c) | Explain how missing data were addressed | 11 |
| Statistical methods | [#12d](https://www.goodreports.org/reporting-checklists/strobe-cross-sectional/info/#12d) | If applicable, describe analytical methods taking account of sampling strategy | 11 |
| Statistical methods | [#12e](https://www.goodreports.org/reporting-checklists/strobe-cross-sectional/info/#12e) | Describe any sensitivity analyses | N/A |
| **Results** |  |  |  |
| Participants | [#13a](https://www.goodreports.org/reporting-checklists/strobe-cross-sectional/info/#13a) | Report numbers of individuals at each stage of study—eg numbers potentially eligible, examined for eligibility, confirmed eligible, included in the study, completing follow-up, and analysed. Give information separately for for exposed and unexposed groups if applicable. | 11 & 12 |
| Participants | [#13b](https://www.goodreports.org/reporting-checklists/strobe-cross-sectional/info/#13b) | Give reasons for non-participation at each stage | 11 & 12 |
| Participants | [#13c](https://www.goodreports.org/reporting-checklists/strobe-cross-sectional/info/#13c) | Consider use of a flow diagram | N/A |
| Descriptive data | [#14a](https://www.goodreports.org/reporting-checklists/strobe-cross-sectional/info/#14a) | Give characteristics of study participants (eg demographic, clinical, social) and information on exposures and potential confounders. Give information separately for exposed and unexposed groups if applicable. | 13 |
| Descriptive data | [#14b](https://www.goodreports.org/reporting-checklists/strobe-cross-sectional/info/#14b) | Indicate number of participants with missing data for each variable of interest | 11 &12 |
| Outcome data | [#15](https://www.goodreports.org/reporting-checklists/strobe-cross-sectional/info/#15) | Report numbers of outcome events or summary measures. Give information separately for exposed and unexposed groups if applicable. | 12 |
| Main results | [#16a](https://www.goodreports.org/reporting-checklists/strobe-cross-sectional/info/#16a) | Give unadjusted estimates and, if applicable, confounder-adjusted estimates and their precision (eg, 95% confidence interval). Make clear which confounders were adjusted for and why they were included | 12, 13, 14, 15, 16, 17 |
| Main results | [#16b](https://www.goodreports.org/reporting-checklists/strobe-cross-sectional/info/#16b) | Report category boundaries when continuous variables were categorized | 12, 13, 14, 15, 16 |
| Main results | [#16c](https://www.goodreports.org/reporting-checklists/strobe-cross-sectional/info/#16c) | If relevant, consider translating estimates of relative risk into absolute risk for a meaningful time period | 12, 13, 14, 15, 16, 17 |
| Other analyses | [#17](https://www.goodreports.org/reporting-checklists/strobe-cross-sectional/info/#17) | Report other analyses done—e.g., analyses of subgroups and interactions, and sensitivity analyses | N/A |
| **Discussion** |  |  |  |
| Key results | [#18](https://www.goodreports.org/reporting-checklists/strobe-cross-sectional/info/#18) | Summarise key results with reference to study objectives | 17 & 18 |
| Limitations | [#19](https://www.goodreports.org/reporting-checklists/strobe-cross-sectional/info/#19) | Discuss limitations of the study, taking into account sources of potential bias or imprecision. Discuss both direction and magnitude of any potential bias. | 18 |
| Interpretation | [#20](https://www.goodreports.org/reporting-checklists/strobe-cross-sectional/info/#20) | Give a cautious overall interpretation considering objectives, limitations, multiplicity of analyses, results from similar studies, and other relevant evidence. | 17 & 18 |
| Generalisability | [#21](https://www.goodreports.org/reporting-checklists/strobe-cross-sectional/info/#21) | Discuss the generalisability (external validity) of the study results | 14 |
| **Other Information** |  |  |  |
| Funding | [#22](https://www.goodreports.org/reporting-checklists/strobe-cross-sectional/info/#22) | Give the source of funding and the role of the funders for the present study and, if applicable, for the original study on which the present article is based | N/A |

Notes:

- 7: 4, 5, 6, 7, 8, 9, & 10
- 12a: 12, 13, 14, 15, & 16
- 12b: 12, 13, 14, 15, 16
- 16a: 12, 13, 14, 15, 16, 17
- 16b: 12, 13, 14, 15, 16
- 16c: 12, 13, 14, 15, 16, 17
- The STROBE checklist is distributed under the terms of the Creative Commons Attribution License CC-BY. This checklist was completed on 26. June 2022 using <https://www.goodreports.org/>, a tool made by the [EQUATOR Network](https://www.equator-network.org) in collaboration with [Penelope.ai](https://www.penelope.ai)
